# Silicone Loss During Histological Preparation of Breast Implant Tissue From Capsular Contracture, Quantified by Stimulated Raman Scattering Microscopy

**DOI:** 10.1002/jbio.202400415

**Published:** 2024-12-23

**Authors:** Robert W. Schmidt, Erik de Bakker, Freek Ariese

**Affiliations:** ^1^ LaserLaB Vrije Universiteit Amsterdam Amsterdam Netherlands; ^2^ Department of Molecular Cell Biology and Immunology, and Plastic, Reconstructive and Hand Surgery Amsterdam UMC, Location VUMC Amsterdam Netherlands

**Keywords:** capsular contracture, silicone, stimulated Raman scattering, tissue preparation

## Abstract

Breast augmentations, commonly performed for aesthetic or medical reasons, often use silicone (polydimethylsiloxane [PDMS]) implants. Some patients develop complications like capsular contracture, where scar tissue forms around the implant. Previously, we used stimulated Raman scattering (SRS) microscopy to detect and quantify silicone in stained capsule tissue, finding a correlation between silicone amount and contracture severity. However, we suspected silicone loss during histological preparation, which includes multiple steps like formalin fixation and paraffin embedding. In this study, we assessed silicone loss by comparing adjacent tissue samples from the same capsule: one prepared conventionally and the other snap‐frozen. SRS microscopy revealed that snap‐frozen samples had roughly five times more silicone, indicating significant silicone loss during conventional preparation. Thus, measuring silicone in histologically prepared samples likely underestimates PDMS content.

## Graphical abstract

Two histological methods, snap‐freezing and paraffin‐embedding, were used on adjacent capsular tissue slices from silicone breast implant patients. Snap‐frozen samples revealed silicone levels approximately five times higher compared to paraffin‐embedded counterparts, suggesting that the latter may underestimate actual silicone content due to losses during preparation.

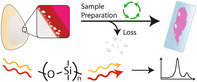

## 1 Introduction

Breast implant surgery is one of the most common plastic surgery performed worldwide, with an estimated 1.7 million procedures in 2021 and 2.2 million in 2022, according to self‐reported numbers by surgeons surveyed by the American Society of Plastic Surgeons [1, 2]. These augmentations are performed by fat transfer or through silicone implants filled with saline or silicone gel [2].

Complications after breast implant surgery have been the subject of substantial academic and public interest. They include but are not limited to, capsular complaints (contracture), breast implants–associated anaplastic large cell lymphoma, and breast implant illness [3, 4]. The implant itself, or bleeding/leaking of silicone into the surrounding tissue is thought to play a role in all these conditions [4, 5, 6]. It is now common practice to advise revision or replacement of the implants at regular intervals [7].

A selective and sensitive detection technique for silicone in tissue is essential for research into the role of silicone in the pathophysiological processes involved in all the silicone‐associated complications of breast implant surgery. Silicone gel implants are made of medical‐grade polydimethylsiloxane (PDMS), often called silicone, which is a chemically inert and thermally stable material [5]. The implant hull consists of elastic silicone rubber, while the filling is made of viscous silicone gel. PDMS molecules are made up of an alternating backbone chain of inorganic silicon (Si) and oxygen, with two methyl groups attached to each Si atom. The length of the polymer chain also influences the viscosity of the PDMS. The precise silicone recipe and the production process are closely guarded secrets by each manufacturer. However, the rough production process is described in the literature [5, 8, 9]. Implant shells are produced by immersing an implant‐shaped template for several seconds in a bath of liquid silicone that also contains fillers that increase the stiffness, electrical conductivity or radiopacity of the shell. Subsequently, the evenly coated templates are cured in a laminar flow oven at high temperatures, which creates a silicone elastomer shell with highly 3D cross‐linked polymers. This is repeated several times until the implant shell consists of 4–10 layers, depending on the manufacturer's specification. Research has shown that implants with a textured surface are less prone to developing stiff fibrous capsules around the implant [10]. A rough implant surface is created by dipping the implant in salt crystals between the silicone bath and the curing step. After the curing process, the salt crystals are removed by washing the implant, leaving a textured surface behind. The implants are filled with PDMS of different viscosities. The foundation structure is given by a lightly cross‐linked silicone gel, which is swollen by short‐chain silicone fluids to give the implant the desired cohesiveness. This means that the silicone fluid can move freely within the lightly cross‐linked silicone network. To retain silicone fluid within the implant, the inside of the implant shells is coated with silicone rubbers that contain phenyl (C_6_H_5_) or trifluoropropyl (CF_3_CH_2_CH_2_) groups. However, cases in which the coating does not fully prevent the bleeding of PDMS through pores in the silicone rubber shell are known [11].

Various analysis techniques exist that can detect the PDMS polymer or its main component, Si in biopsies. For example, elemental Si was quantified in the breast and capsular tissue of silicone implants by inductively coupled plasma atomic emission spectroscopy [12]. This sensitive method is capable of determining trace element concentrations, but it is also a destructive method, since tissues are digested with acid for analysis, thus losing spatial information about the silicone locations and distribution. Energy‐dispersive x‐ray (EDX) analysis has been used as a qualitative method to identify Si. However, it was used only at a few locations in histologically prepared tissues to confirm the presence of elemental Si [13, 14]. Kappel et al. [11, 13] combined EDX with a dye called Oil Red O to stain PDMS in organs and breast capsules and qualitatively demonstrated with EDX that the Oil Red O dye stained silicone particles. However, this dye also binds to neutral fats, fatty acids and triglycerides and is therefore not a silicone gel–specific dye [15, 16]. Infrared microspectroscopy has been used to scan implant capsules for PDMS but has a physically limited spatial resolution [17]. Furthermore, infrared light is absorbed by glass, thus making the method not compatible with standard histopathology microscopy glass slides. Instead, tissues must be prepared on costly CaF_2_ substrates for IR measurements.

As an alternative to IR spectroscopy, Raman spectroscopy is an analytical technique that measures the vibrational energies in a molecule. This is done by sending a laser with a narrow wavelength onto a sample; the laser photons can be absorbed by the molecule or re‐emitted at the same wavelength, which is called elastic scattering. In rare cases, the photon is scattered at another wavelength (inelastic scattering), and the energy shift of the scattered photons corresponds to the vibrational modes in the molecule, which are unique for every molecule and can be used to identify compounds. Although Raman spectroscopy is a nondestructive method able to measure tissues fixed on standard microscopy glass slides, it is also a slow method, especially when scanning tissues at submicrometer resolution. Stimulated Raman Scattering (SRS) microscopy overcomes this shortfall by increasing the measuring speed by a factor of 3–5 orders of magnitude, making it possible to scan a tissue for PDMS within a reasonable time [18]. However, instead of acquiring a detailed Raman spectrum for each location, narrow‐band SRS can scan only at one wavenumber at a time and is therefore limited to carefully chosen wavenumbers. SRS is a technique that uses two pulsed lasers in the near‐infrared region; one is used to excite the molecule to a virtual energy state (Pump), while the other laser is used to stimulate a specific vibrational transition in the molecule (Stokes). A stimulated emission signal is generated when the difference in frequency between those lasers matches that of a vibration in the molecule of interest at the focal spot of the two lasers. The Stokes laser gains energy (intensity) through the stimulated emission of photons, whereas the pump laser loses energy. This energy transfer between the lasers is only a very small fraction of the intensity but can be measured by modulating one of the lasers (in our case the Stokes beam) and using a lock‐in amplifier for detection of the intensity fluctuations in the pump signal. [19, 20] These stimulated transitions result in a stronger signal, which means that the exposure time per pixel can be reduced to microseconds instead of seconds with conventional spontaneous Raman scattering. Importantly, the SRS signal can only be generated if both pulsed lasers overlap in time and space within the sample, which improves the measurable z‐resolution in the sample dramatically [21]. Furthermore, SRS distinguishes itself from spontaneous Raman spectroscopy by suppressing the fluorescence signal, which can arise from sources such as glass, glue or autofluorescence, and often obscures the Raman peaks in conventional Raman mapping [22].

Silicone fluids are highly soluble in hydrocarbon solvents (toluene, xylene), mineral spirits and chlorinated hydrocarbons, but are not soluble in water (hydrophobic) [23]. While highly cross‐linked silicones like gels and rubbers remain undissolved, high‐solubility solvents such as xylene can penetrate the cross‐linked polymer matrix, leading to swelling and potentially extracting uncross‐linked PDMS fluids. The tissue comes into contact with different solvents during the paraffin embedding process (fixation, dehydration and defatting). In addition, the sliced tissues are transferred onto a microscopy slide through a flotation water bath, which leads to the belief that silicone could be washed off the tissue during each of these processing steps. Kappel et al. [13] already suggested in their research that paraffin preparation can dissolve some of the silicone. However, the extent of silicone loss during fixation and paraffin embedding was not quantified. Fixation serves to immobilize complexes, cells and tissues for further analysis. Typically, formaldehyde is used to crosslink tissues by forming covalent bonds with amino groups in proteins or DNA. This process involves the formation of a methylol intermediate, which converts into a Schiff base and then forms a methylene bridge between two amino acids, establishing a crosslinked structure [24]. Despite its efficacy in protein/DNA crosslinking, formaldehyde is unlikely to crosslink with PDMS due to the absence of strong nucleophiles, such as amino groups. Consequently, formaldehyde cannot chemically bind PDMS to the tissue, but the crosslinked tissue might physically retain larger PDMS particles.

In this study, we determine the extent of possible silicone loss during standard histological sample preparation; formalin fixation, paraffin embedding and cutting. We selected tissues that we expected to contain uniformly distributed amounts of deposited silicone from bleeding implants. In a previous study, we already found a correlation between silicone concentration in capsule tissue and the severity of capsular contracture by comparing samples from patients with bilateral implants but unilateral contracture [4]. For the current study, we selected capsule tissue from patients with severe capsular contracture (Baker IV) and processed the tissues in two ways. One piece of the capsule was prepared by paraffin embedding, while the adjacent piece of the capsule was prepared by snap freezing. Snap freezing avoids the many washing steps of paraffin embedding in which silicone particles could be lost. We measured both paraffin‐embedded and snap‐frozen tissues with SRS microscopy because of its non‐destructive nature, fast acquisition and high chemical specificity to determine silicone levels in the tissues. From the SRS images, the silicone levels were quantitatively determined at micrometer resolution.

## 2 Methodology

### 2.1 SRS Microscopy

SRS microscopy images were captured with a custom‐built system as previously described [22] and can be found in the Supporting Information 1.1. Two scans per tissue were obtained, one at a wavenumber that exhibits a strong Raman peak from the methyl groups (CH_3_ stretch) of PDMS and one where no PDMS signal is present. The corresponding wavenumbers are 2905 cm^−1^ for silicone and 2933 cm^−1^ for the ‘tissue background’ [22]. The pixel step size for all measurements was 1.62 μm and the powers under the microscope were set at 7 mW for the Pump and 14 mW for the Stokes beam. This power ratio of 1:2 for the Pump and Stokes was recommended by Moester et al. [25]. The images were acquired with a pixel dwelltime of 100 μs which allows to scan one square centimeter (cm^2^) in about 1 h at one wavenumber. A detailed description of the SRS setup and the data processing is reported in the SI.

### 2.2 Tissue Preparation Protocol

Adjacent samples were cut from a representative region of the entire explanted capsules and subsequently either taken for regular tissue preparation in paraffin or snap‐frozen in liquid nitrogen and stored at −80°C. Samples intended for regular preparation were fixed in a 4% formaldehyde solution for at least 24 h. At the pathology department, these samples were processed routinely using a TissueTek VIP 6 (Sakura). In short, this involves a dehydration cycle with increasing ethanol concentration; 70%, 80%, 96% and three iterations of 100% ethanol, each 1 h long at 35°C; next, a cleaning cycle of three iterations with xylene of 1 h long at 35°C; finally, three‐hour impregnating cycles at 63°C in paraffin. Samples were then embedded in paraffin before 5 to 30 μm sections were cut using a microtome. The sections were transferred to a glass slide. The samples were then used without further processing.

Snap‐frozen samples were cut into 5 or 50 μm sections using a cryostat (CryoStar NX70, Fisher Scientific). After air‐drying for 10 min, they were covered using cryomatrix embedding resin (Epredia, Fisher Scientific) and fixed between a glass slide and a coverslip.

### 2.3 Ethics

Clinical grading, the explantation of implants and the collection of capsules were performed by an experienced plastic surgeon (FBN) and samples were included only after oral informed consent. Tissue samples were collected in compliance with the ‘Code for Proper Secondary Use of Human Tissue’ as formulated by the Dutch Federation of Medical Scientific Organizations. This study was performed per the Declaration of Helsinki and the guidelines for Good Clinical Practice.

## 3 Results and Discussion

The tissues of two Baker IV capsules (severe contraction) were divided into three pairs and adjacent slices were prepared snap‐frozen or embedded in paraffin and microtomed. In total, 31 tissues were analysed, of which 14 were snap frozen and 17 were embedded in paraffin. Using SRS microscopy, silicone particles were found in 12 of 14 snap‐frozen slices and 9 of 17 paraffin‐embedded tissue slices.

**FIGURE 1:**
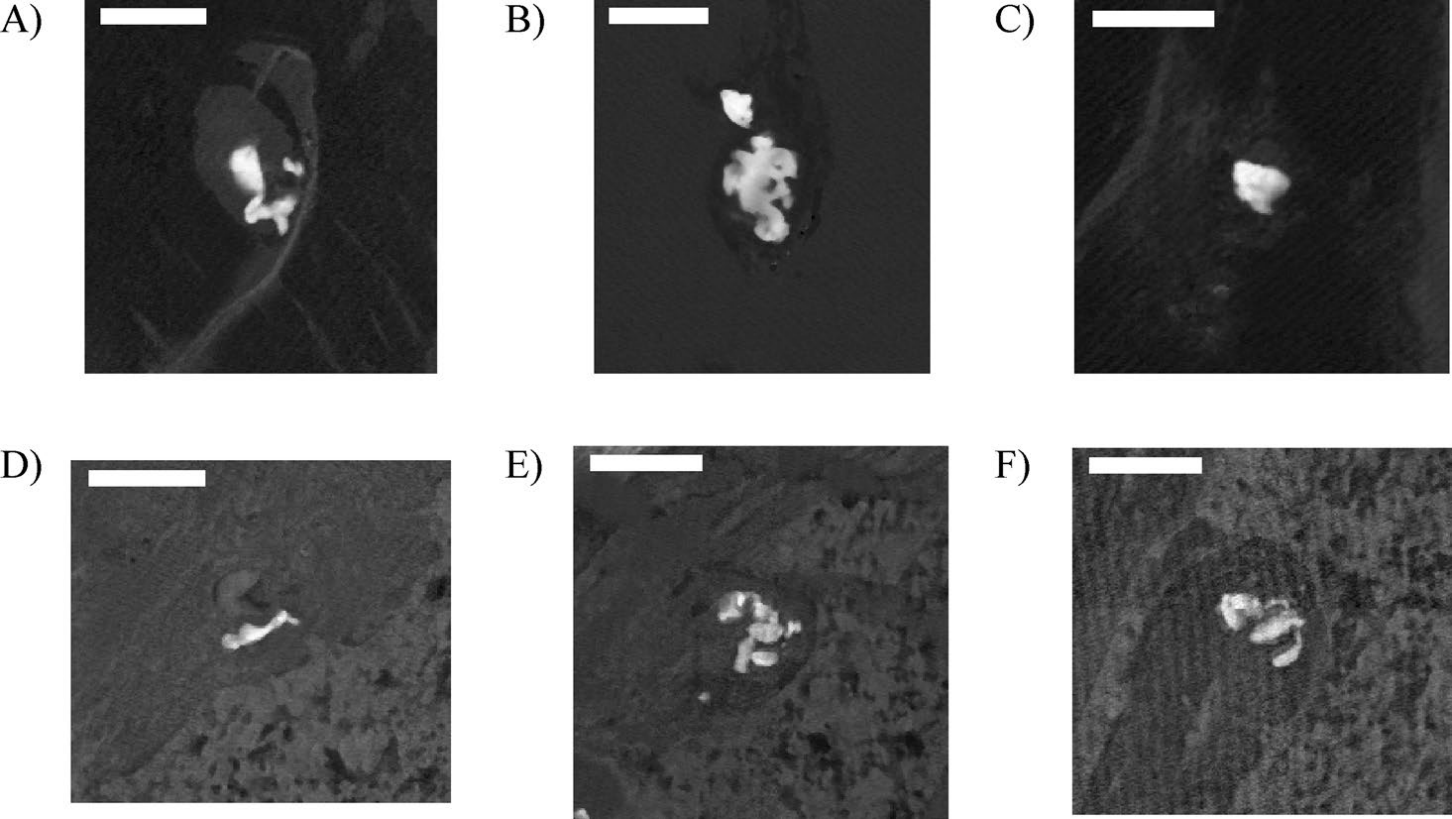
Selection of silicone‐specific abundance maps acquired by SRS microscopy of selected locations from the third biopsy. Silicone particles are visible as bright objects in the snap‐frozen (A–C) and paraffin‐embedded (D–F) tissue slices. Furthermore, in the paraffin‐embedded images, the paraffin matrix and tissue morphology are visible. Scale bar 100 μm.

During the inspection of the generated PDMS abundance maps, it became clear that the silicone particles were located close to the edge on one side of the tissue, which had been adjacent to the silicone implant (Figure 1). This is also illustrated in Figure S1. Furthermore, the particles in paraffin‐embedded slices seemed to be more clustered than in snap‐frozen samples. Figure 1 shows typical example shapes of selected silicone particles in snap‐frozen (A–C) and paraffin‐embedded (D–F) samples. These silicone particles can be described as small single thin needle–shaped fibers with rounded edges or large objects that appear to consist of multiple intertwined particle clusters. Furthermore, in the paraffin‐embedded images, the paraffin and tissue are also visible, showing that the silicone particles are enclosed by collagen fibers next to the edge of the tissue. In the images of Figure S1, the paraffin is located at the bottom right.

**FIGURE 2:**
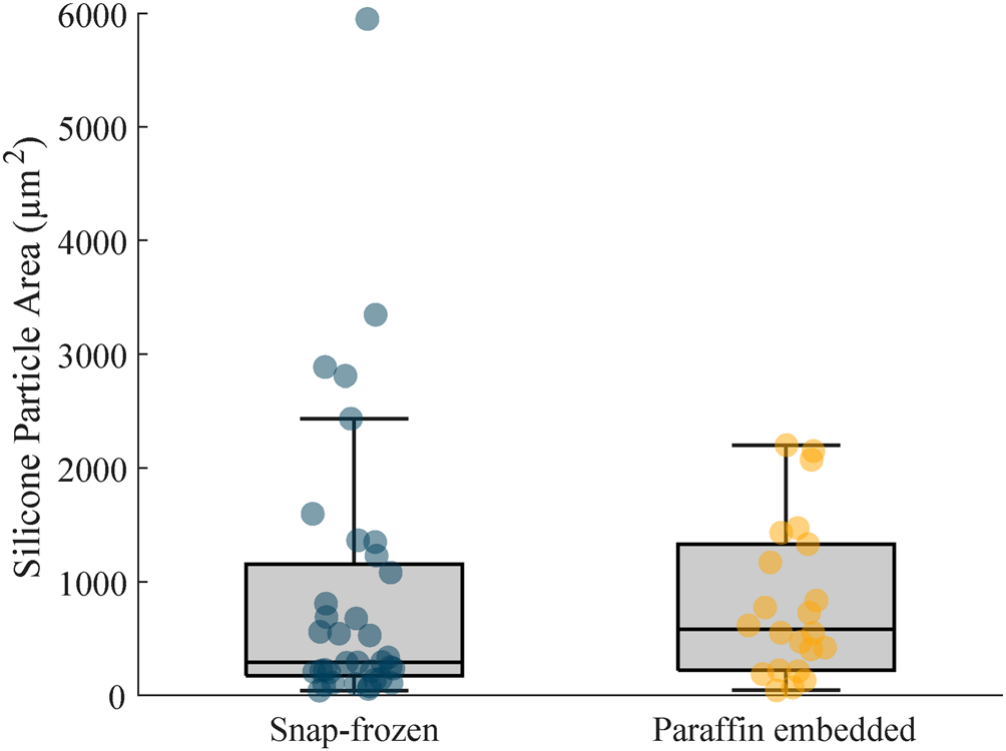
Particle sizes of silicone found in breast implant capsules that were histologically prepared by snap‐freezing and paraffin embedding. The median particle area in snap‐frozen tissues is 291 μm^2^ and in paraffin‐embedded is 582 μm^2^.

In total, 36 particles were detected in 14 snap‐frozen samples with a median size of 291 μm^2^, a lower quartile of 174 μm^2^ and an upper quartile of 1155 μm^2^, as seen in Figure 2. The smallest particle had a size of 40 μm^2^ and the largest particle had a size of 5949 μm^2^. The paraffin‐embedded sample contained 22 particles with a median size of 582 μm^2^, the lower quartile was at 220 μm^2^ and the upper quartile was at 1331 μm^2^. The smallest particle found had a size of 43 μm^2^ while the largest had a size of 2202 μm^2^. Expressing the number of particles found in tissues per area, we found 31.0 particles per cm^2^ in snap‐frozen tissues and 6.8 particles per cm^2^ in paraffin‐embedded tissues.

**FIGURE 3:**
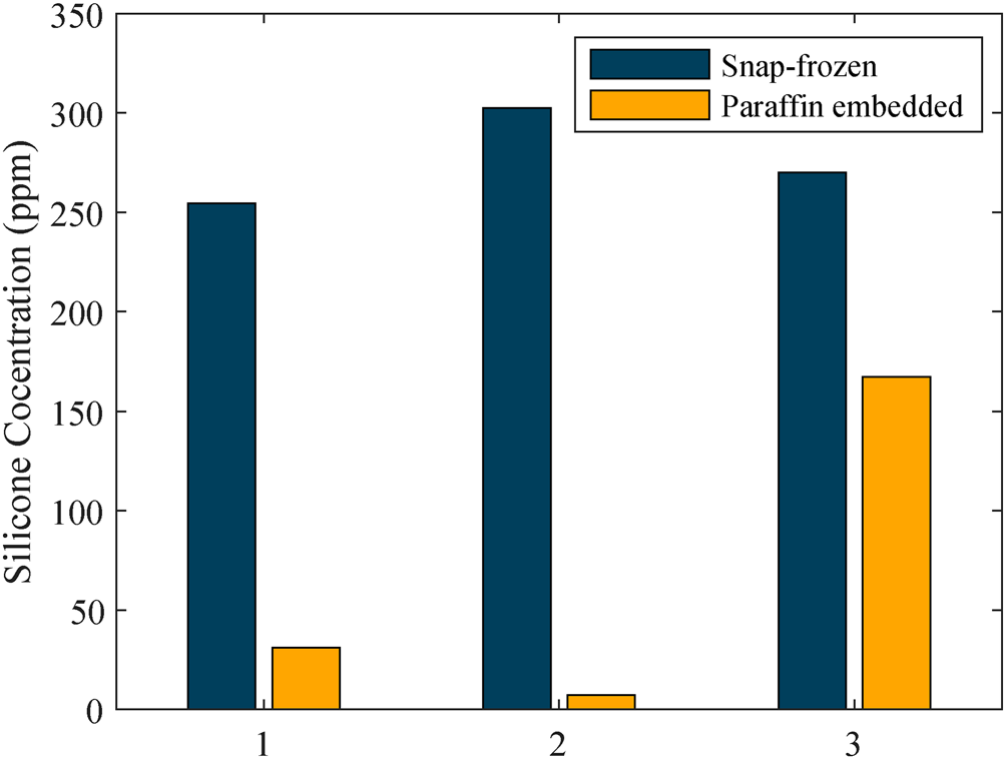
Concentration of silicone particles (area relative to the total tissue area) in snap‐frozen prepared and paraffin‐embedded tissues from three locations in Baker IV grade capsules.

Due to the different sample sizes in our study, we quantified the silicone concentration in relation to the total area of the tissue (excluding the fixation matrix/paraffin). This relative area is expressed here in ppm: μm^2^ of silicone area per mm^2^ of tissue area. In the three snap‐frozen tissues samples, an average area silicone concentration of 254, 302 and 270 ppm was found, whereas in paraffin‐embedded tissues, we detected 31, 7 and 167 ppm of silicone, as seen in Figure 3.

Overall, these results indicate that the snap‐frozen prepared tissues contained many more particles, both smaller and larger, compared to the paraffin‐embedded samples, see Figure 2. Furthermore, it was determined that silicone had a lower concentration in paraffin‐embedded slices when comparing all tissue pairs, see Figure 3. This would suggest that small and large particles were washed out or reduced in size during the multiple processing steps of paraffin‐embedding, with few medium‐sized particles remaining in the tissue.

It should be mentioned that, given the study design based on adjacent samples, we assumed an even distribution of particles within the sample pairs; nevertheless, silicone ‘bleeding’ or shedding is an uncontrollable naturally occurring process that could have led to uneven silicone concentrations between the analyzed tissue pairs.

SRS microscopy is a fast and chemically specific method to identify polymers such as PDMS in samples. The spatial (*x*, *y*) resolution is 0.67 μm [26] which makes it possible to measure the size and distribution of silicone debris in the samples. However, the SRS signal is only generated in the focal overlap between the Stokes and Pump beam, which we had previously determined to have a *z* height of 2.6 μm [27]. The particles above or below the focal volume do not contribute to the SRS signal and will not be observed. Furthermore, paraffin also has a weak Raman peak at 2905 cm^−1^, the same wavenumber used for silicone‐specific imaging. Consequently, scans of paraffin‐embedded samples at this wavenumber show a better contrast, revealing not only silicone particles but also the morphology of the tissue (Figures 1 and S1). This contrast‐enhancing effect may become negligible after further processing and staining. As fluid and difficult to completely fix as silicone is it should be assumed that the cryotechnique will also lead to an unknown loss of silicone out of the tissue, as this kind of diffusion will probably start even during surgery. Additional processing steps could be evaluated to reduce the amount of silicone loss even further. In this study, we report the area of the particles compared to the area of the tissue to overcome uneven sample sizes.

During the SRS acquisition, we encountered transient absorption artifacts that showed up as individual bright pixels in the binary silicone image. To avoid falsely classifying these as silicone particles, we applied a connected component filter that removed particles with fewer than five connected pixels, corresponding with a lower size limit of six pixels (15.7 μm^2^). Furthermore, we used a GUI to manually check the classified silicone particles, which reduced the falsely classified silicone particle. However, this is a quite laborious method to overcome false positives due to subtraction artefacts, and could be solved by scanning the sample with a third wavelength for silicone or background, which in turn would increase the scanning time by a factor of 1.5.

The most striking observation that emerged from the analysis based on the particle sizes and distribution was that large particles become smaller during histological preparation because of the partial removal of PDMS, whereas small particles dissolve and are no longer detected. This would explain the different concentration of silicone particles described in Figure 2. Given silicone's solubility in xylene, it is likely that small particles or fluids were washed away during the xylene treatment.

## 4 Conclusion

Histopathological sample preparation by paraffin embedding, formalin fixation and staining is the gold standard in the pathological field. However, during this procedure, the tissues are exposed to mechanical stress and washed several times with non‐polar solvents to fixate, defat and dehydrate the tissue. Tissues containing unincorporated foreign material like PDMS from silicone implants are prone to losing these particles since the washing steps can remove or dissolve those particles.

The tissues of two Baker IV capsules (severe contraction) were divided into three pairs, and adjacent slices were prepared snap‐frozen or embedded in paraffin and then analysed. On average, more silicone particles were found in snap‐frozen than in paraffin‐embedded tissues. Due to the different sample sizes in our study, we quantified the silicone area relative to the total area of the analysed tissues. In the snap‐frozen prepared tissues, we found concentrations of silicone that were on average more than five times higher than in the paraffin‐embedded tissue.

This research showed a loss of silicone particles during paraffin embedding that is commonly used before staining the tissue (e.g., H&E or MORO for silicone). Thus, a severe underestimation of the level of silicone debris is expected when paraffin‐embedded samples are used to determine the extent of PDMS leakage.

## Author Contributions

**R.W.S.:** conceptualization, methodology, software, formal analysis, investigation and writing – original draft preparation. **E.B.:** conceptualization, resources, writing – review and editing. **F.A.:** conceptualization, writing – review and editing, supervision and funding acquisition.

## Conflicts of Interest

The authors declare no conflicts of interest.

## Supporting information


**Table S1.** Overview of silicone particles found in snap‐frozen prepared tissues.
**Table S2**. Overview of silicone particles found in paraffin‐embedded tissues.

**Figure S1.** Stimulated Raman microscopy images showcasing 15 silicone particles within paraffin‐embedded tissue from Location 3. Two images are shown per particle. The left images depict the SRS scan at the silicone wavenumber, with silicones appearing as bright pixels. Paraffin, primarily located in the bottom right of the images, appears as grayish dabs, while the tissue is mainly darker. This contrast highlights the tissue morphology, indicating that the silicones are primarily located at the tissue’s edge. The right image presents the subtracted silicone‐specific image with a silicone mask overlaid as transparent red pixels. Scale bar: 100 μm.

## Data Availability

The data that support the findings of this study are available from the corresponding author upon reasonable request.
