# Supplementary material for: Silicone Loss During Histological Preparation of Breast Implant Tissue From Capsular Contracture, Quantified by Stimulated Raman Scattering Microscopy
Source: J Biophotonics. 2024 Dec 23;18(2):e202400415. doi: 10.1002/jbio.202400415 (PMC11793946; doi:10.1002/jbio.202400415)
Supplement: Supplementary file 1 — Table S1. Overview of silicone particles found in snap‐frozen prepared tissues. Table S2. Overview of silicone particles found in paraffin‐embedded tissues. [file JBIO-18-e202400415-s001.docx]

## 1 Supplementary Information

### 1.1 Stimulated Raman Scattering Microscopy setup

A picosecond Lumera Plecter Duo Nd:YAG laser with a 1064 nm and a frequency-doubled 532 nm output was used. The 532 nm beam was used to pump a Levante Emerald Optical Parametric Oscillator (OPO), while the output 1064 nm beam was sent onto a delay stage and to an Acousto-Optical Modulator at 3.636 MHz from EQ Photonics GmbH. The OPO (Pump beam) was tuned so that the photon energy difference with the fixed 1064 nm beam (Stokes) would correspond with the targeted molecular vibration. Then both beams were spatially overlaid with the help of a dichroic mirror and sent to a Zeiss Examiner 7MP laser scanning microscope. We used a C-achroplan W 32x water immersion objective with a numerical aperture of 0.85 and a water immersion condenser with a numerical aperture of 1.2. The 1064 nm beam was blocked by a filter and the pump beam intensity was measured to reveal the stimulated Raman loss signal. For detection, we used a DET36A photodetector from Thorlabs, and the signal was demodulated with a lock-in amplifier from Zurich Instruments. SRS scans were acquired through Zeiss’s proprietary ZEN2011 microscope software with a step size of 1.62 µm, a pixel dwell time of 100 µs.

### 1.2 Data processing

Data processing of the acquired SRS images was performed in MATLAB R2021a. The tissue area was determined by creating a tissue-specific mask by applying a graph cut function that classifies the tissue and the background pixels. The vacuoles in the tissue were also counted as tissue area, and the mask was closed with MATLAB’s fill-hole function. Burn or transient absorption artefacts from the SRS measurements can hinder the data analysis because they appear in the SRS images as extremely dark or bright pixels. These pixels are identified by calculating the three-times standard variation of the pixel intensity and are removed by replacing them with median smoothed pixels from the neighborhood (3x3). Subsequently, the pixel intensities of the background SRS image were adjusted to that of the silicone image intensity to compensate for variations in laser power between the image acquisitions; see the images in Figure S1 left. A silicone-specific image was created by subtracting the background scan from the silicone scan, and outlier pixels were removed with the same method as the burn and transient absorption artefacts (right images of Fig. S1. A threshold was applied to the image to create a binary silicone mask of the brightest pixels. Small particles in the binary mask that contained less than six connected pixels (min area 16 µm^2^) were seen as possible artefacts and removed from the silicone mask (Fig. S1 red overlay on the right images). Each classified silicone particle was then visually inspected to reduce false positives by considering the shape of the particles, the pixel intensities in the SRS images, and the locations of the particles.

Figure S1: Stimulated Stimulated Raman microscopy images showcasing 15 silicone particles within paraffin-embedded tissue from location 3. Two images are shown per particle. The left images depict the SRS scan at the silicone wavenumber, with silicones appearing as bright pixels. Paraffin, primarily located in the bottom right of the images, appears as grayish dabs, while the tissue is mainly darker. This contrast highlights the tissue morphology, indicating that the silicones are primarily located at the tissue’s edge. The right image presents the subtracted silicone-specific image with a silicone mask overlaid as transparent red pixels. Scale bar: 100 µm.

### 1.3 Overview of particles per tissue

Table S1 and S2 show the number and area of silicone particles found in frozen frozen snap tissues and paraffin-embedded tissues, respectively. Each row corresponds to an analyzed tissue slice.

| Capsule location | Tissue number | Number of particles in tissue | Area of silicone particles µm^2^ | Area of tissue in mm^2^ |
| --- | --- | --- | --- | --- |
|  | 1 | 0 | 0 | 5.98 |
| 1 | 2 | 10 | 7578 | 4.71 |
| 1 | 3 | 1 | 223 | 7.31 |
| 1 | 4 | 1 | 56 | 3.55 |
| 1 | 5 | 4 | 737 | 6.77 |
| 1 | 6 | 2 | 525 | 7.53 |
| 2 | 1 | 3 | 428 | 6.84 |
| 2 | 2 | 5 | 6147 | 14.90 |
| 3 | 1 | 2 | 3095 | 9.59 |
| 3 | 2 | 1 | 689 | 10.02 |
| 3 | 3 | 0 | 0 | 3.24 |
| 3 | 4 | 2 | 1708 | 14.45 |
| 3 | 5 | 2 | 7030 | 12.47 |
| 3 | 6 | 3 | 3241 | 8.64 |

Table S1: Overview of silicone particles found in snap frozen prepared tissues.

| Capsule location | Tissue number | Number of particles in tissue | Area of silicone particles µm^2^ | Tissue area in mm^2^ |
| --- | --- | --- | --- | --- |
|  | 1 | 2 | 2143 | 25.63 |
| 1 | 2 | 1 | 550 | 9.38 |
| 1 | 3 | 0 | 0 | 25.71 |
| 1 | 4 | 0 | 0 | 24.64 |
| 1 | 5 | 1 | 773 | 27.73 |
| 1 | 6 | 2 | 1518 | 21.09 |
| 1 | 7 | 0 | 0 | 26.40 |
| 2 | 1 | 0 | 0 | 14.25 |
| 2 | 2 | 0 | 0 | 14.20 |
| 2 | 3 | 0 | 0 | 16.39 |
| 2 | 4 | 0 | 0 | 13.07 |
| 2 | 5 | 1 | 614 | 13.73 |
| 2 | 6 | 0 | 0 | 15.16 |
| 3 | 1 | 8 | 5125 | 12.35 |
| 3 | 2 | 4 | 3988 | 17.47 |
| 3 | 3 | 1 | 1472 | 22.82 |
| 3 | 4 | 2 | 1850.88 | 21.77 |

Table S2: Overview of silicone particles found in paraffin embedded tissues.
