# Supplementary figures and images for: Silicone Loss During Histological Preparation of Breast Implant Tissue From Capsular Contracture, Quantified by Stimulated Raman Scattering Microscopy
Source: J Biophotonics. 2024 Dec 23;18(2):e202400415. doi: 10.1002/jbio.202400415 (PMC11793946; doi:10.1002/jbio.202400415)

Silicone

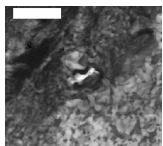

Subtracted+Mask

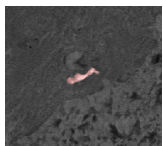

Silicone

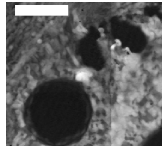

Subtracted+Mask

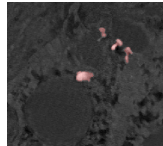

Silicone

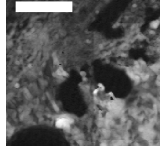

Subtracted+Mask

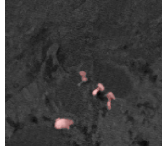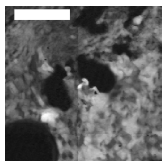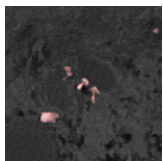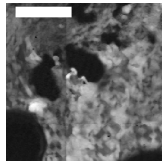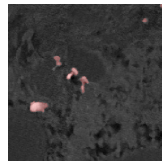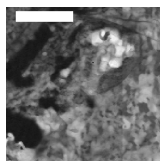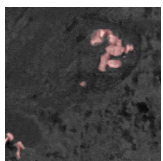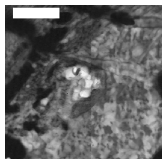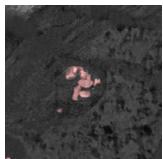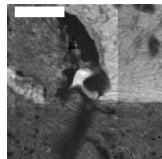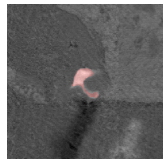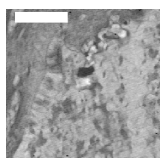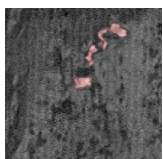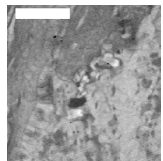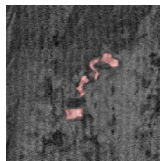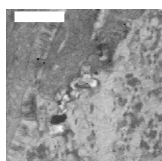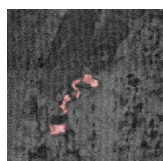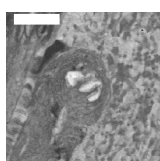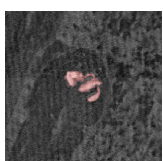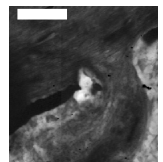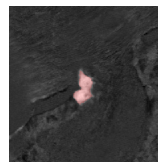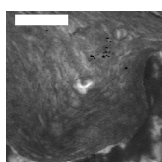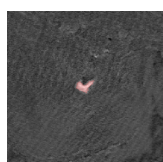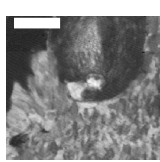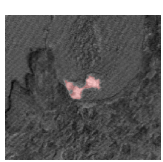

Supplement: Supplementary file 2 — Figure S1. Stimulated Raman microscopy images showcasing 15 silicone particles within paraffin‐embedded tissue from Location 3. Two images are shown per particle. The left images depict the SRS scan at the silicone wavenumber, with silicones appearing as bright pixels. Paraffin, primarily located in the bottom right of the images, appears as grayish dabs, while the tissue is mainly darker. This contrast highlights the tissue morphology, indicating that the silicones are primarily located at the tissue’s edge. The right image presents the subtracted silicone‐specific image with a silicone mask overlaid as transparent red pixels. Scale bar: 100 μm. [file JBIO-18-e202400415-s002.pdf]
